# Free breathing myocardial perfusion data sets for performance analysis of motion compensation algorithms

**DOI:** 10.1186/2047-217X-3-23

**Published:** 2014-11-11

**Authors:** Gert Wollny, Peter Kellman

**Affiliations:** 1 Biomedical Imaging Technologies, ETSI Telecomunicación, Universidad Politécnica de Madrid, Avenida Complutense 30, 28040 Madrid, Spain; 2 Ciber BBN, Zaragoza, Spain; 3 Laboratory of Cardiac Energetics, National Heart, Lung and Blood Institute, National Institutes of Health, DHHS, Bethesda, MD, USA

**Keywords:** Heart, Image registration, Motion compensation, Perfusion, Validation

## Abstract

**Background:**

Perfusion quantification by using first-pass gadolinium-enhanced myocardial perfusion magnetic resonance imaging (MRI) has proved to be a reliable tool for the diagnosis of coronary artery disease that leads to reduced blood flow to the myocardium. The image series resulting from such acquisition usually exhibits a breathing motion that needs to be compensated for if a further automatic analysis of the perfusion is to be executed. Various algorithms have been presented to facilitate such a motion compensation, but the lack of publicly available data sets hinders a proper, reproducible comparison of these algorithms.

**Material:**

Free breathing perfusion MRI series of ten patients considered clinically to have a stress perfusion defect were acquired; for each patient a rest and a stress study was executed. Manual segmentations of the left ventricle myocardium and the right-left ventricle insertion point are provided for all images in order to make a unified validation of the motion compensation algorithms and the perfusion analysis possible. In addition, all the scripts and the software required to run the experiments are provided alongside the data, and to enable interested parties to directly run the experiments themselves, the test bed is also provided as a virtual hard disk.

**Findings:**

To illustrate the utility of the data set two motion compensation algorithms with publicly available implementations were applied to the data and earlier reported results about the performance of these algorithms could be confirmed.

**Conclusion:**

The data repository alongside the evaluation test bed provides the option to reliably compare motion compensation algorithms for myocardial perfusion MRI. In addition, we encourage that researchers add their own annotations to the data set, either to provide inter-observer comparisons of segmentations, or to make other applications possible, for example, the validation of segmentation algorithms.

## Background

**Figure 1:**
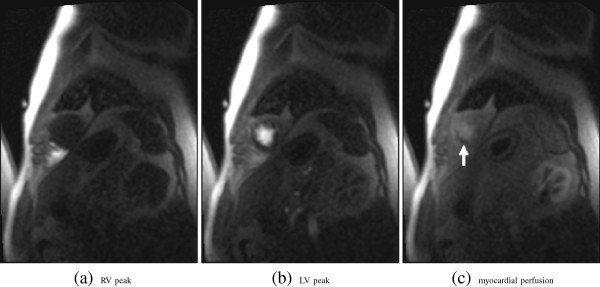
**Example images from a first-pass gadolinium-enhanced myocardial perfusion MRI study (patient 5, stress, apical slice): RV enhacement peak (a), LV enhancement peak (b) and myocardial perfusion (c).** Note, the hypointense region in the perfused myocardium **(c)** indicates a reduction in blood flow, i.e. the medical condition that needs to be quantified for the assessment.

Perfusion quantification by using first-pass gadolinium-enhanced myocardial perfusion magnetic resonance imaging (MRI) has proved to be a reliable tool for the diagnosis of coronary artery disease that leads to reduced blood flow to the myocardium. With a typical imaging protocol, images are usually acquired for 60 seconds and the acquired image series includes some pre-contrast baseline images, with the full cycle of contrast agent first entering the right ventricle (RV), then the left ventricle (LV), and finally perfusing the LV myocardium (Figure 1). In order to quantify blood flow, the image intensity in the myocardium is tracked over time ([1,2]).

In order to perform an automatic assessment of the intensity change over time, no movement should occur between images taken at different times. Electrocardiography (ECG) triggering is used to ensure that the heart is always imaged at the same cardiac phase. However, since the 60 seconds acquisition time span is too long for the average person to hold their breath, breathing movement is usually present in the image series.

To compensate for this breathing motion by image processing methods two problems have to be overcome: The motion to be compensated for itself, and the rather strong intensity change that is induced by the contrast agent. A wide variety of methods have been proposed to eliminate the motion from the images series by image registration. Image registration is the process of finding a transformation that maps on image, the moving or floating image to another image, the template or reference image so as to optimize a measure describing the similarity of these two images.

Some motion compensation methods rely on linear registration only (i.e. only a linear transformation is optimized), for example, translation [3-7], or translation and rotation [8,9]. Note, that in addition to translation and rotation a linear transformation may also comprise scaling and shear.

However, since the breathing movement results in the heart moving within the barely moving chest, the all-over movement pattern is highly non-linear, and masking is needed to extract a region of interest (ROI) around the heart that must be small enough to not contain non-moving body parts but big enough to accommodate the full movement range of the heart itself. In addition, linear registration does not account for the non-linear deformations of the myocardium itself. On the other hand, employing non-linear registration does not require the extraction of a bounding box and it can also compensate for the non-linear deformations of the heart.

To overcome the challenge of changing intensities one can optimize statistical image similarity measures such as mutual information or cross correlation (e.g. [10,11]), or gradient based measures [12,13]. One can minimize the amount of intensity change between images by only running the image registration for image pairs in direct temporal succession, and then align all images to one reference by accumulating the obtained transformations [11,12,14]. However, this accumulation of transformation may also result in the accumulation of small registration errors, and may, therefore, result in considerable large errors in the overall alignment for time steps that are “far away” from the common reference in the temporal succession. An alternative is to model the intensity change and create motion-free images that can then be used as reference images for a following registration step [13,15-20]. Since the motion manifests itself as a high frequency component in intensity change over time it has also been proposed to work in the frequency domain and remove these high frequency components [6]. For detailed review of motion compensation methods that can be applied to myocardial perfusion image series the reader is referred to [21].

A fair comparison of these algorithms requires that common data is used for the validation procedure, but because of the personal nature of the medical data used, its free redistribution is usually impeded by patient privacy concerns.

Here we present a manually segmented series of myocardial perfusion data sets that were acquired with a free breathing protocol, along with the required patient consent, anonymization, and un-linking that makes its free redistribution possible. In addition we give an overview of the provided data, as well as provide results of motion compensation experiments run with the data that replicate work presented elsewhere [13,20]. In these publications the experiments were executed with different data sets that could not be redistributed freely. Finally, we give short descriptions of the applied motion compensation exploiting the quasiperiodicy of the breathing movement (QUASI-P) [13] and motion compensation using independent component analysis and a spline based non-linear registration scheme (ICA-SP) [20] used to run these experiments, including the small enhancements we applied with respect to their original implementation.

## Data description

### Acquisition and data features

In total, anonymized and unlinked data sets of ten patients considered clinically to have a stress perfusion defect are made available. The data was acquired under clinical research protocols approved by the Institutional Review Boards of the National Heart, Lung, and Blood Institute and Suburban Hospital (Bethesda, MD, USA). The patients provided written informed consent, and the analysis and data repository were approved by the NIH Office of Human Subject Research. For each patient, both rest study and stress studies were executed and the perfusion images were acquired by using a free breathing protocol. However, patient six had extremely shallow breathing so that the rest study appears to be acquired with a complete breath hold (i.e. it contains no visible motion), and the stress study only exhibits shallow breathing. For the first patient images were taken in only 45 time steps, and for the remaining nine patients images were taken in 60 time steps. In all cases, three slices (at the base, mid, and apical level) were acquired. Hence, in total 60 2D image series are provided in the data set (two studies for each of the 10 patients with three slices each). An overview of the data sets is given in Table 1.

**Table 1:** Available data sets

**Patient**		**Time steps**	**Breathing regularity**	**Image quality**	**Spatial intensity homogeneity**	**Remarks**
1	Rest	45	3	5	Low	
	Stress		4	5	Low	
2	Rest	60	5	5	Medium	
	Stress		4	4	Medium	
3	Rest	60	5	5	Low	
	Stress		5	4	Low	
4	Rest	60	5	5	Low	
	Stress		5	5	Low	
5	Rest	60	2	5	Medium	
	Stress		4	5	Medium	
6	Rest	60	-	3	High	No movement
	Stress		3	3	High	Shallow breathing
7	Rest	60	5	5	Medium	
	Stress		5	5	Medium	Slow breathing rate
8	Rest	60	5	5	Medium	
	Stress		5	5	Medium	
9	Rest	60	4	4	Medium	
	Stress		5	4	Medium	
10	Rest	60	3	5	Low	
	Stress		3	5	Medium	

The rating (5 = best) for breathing regularity, image quality, and intensity inhomogeneity is subjective and based on visualizing the series as a looping video.

For all patients, a half dose of contrast agent (Gd-DTPA, 0.05 mmol/kg) was administered at 5 ml/s, followed by 20 ml saline flush. The first two time steps of each series comprise proton density weighted images that may be used for intensity inhomogeneity correction (see, e.g., [11]); however, this intensity correction is not considered here. The remaining slices were acquired by using the slew rate – fast low angle shot (SR-FLASH) protocol. For all series TR/TE=2.41/1.06, except for patient 6 (TR/TE=2.43/1.22). The images series were reconstructed to a final matrix size of 256 × 192 (3/4 phase FOV) using zero filling for interpolation. The resulting interpolated spatial pixel resolution is 1.4 mm × 1.4 mm for patients 1–5, 9 and 10, and 1.6 mm × 1.6 mm for the patients 6, 7, and 8. An example of the key perfusion time steps – RV enhancement, LV enhancement, and myocardial perfusion – at the base level is given in Figure 1.

The series images are all provided as anonymized DICOM files and as 8-bit PNG files. A linear mapping from the 16-bit DICOM pixel intensity range to the 8-bit PNG intensity range was applied that maximizes visual quality and the mapping parameters were selected individually. Therefore, the 8-bit data should not be used for measurements that aims at comparing data from different slice series. PNG files are required because the segmentation software [22] used is currently limited to image formats that are directly supported the Android operating system.

### Segmentations

**Figure 2:**
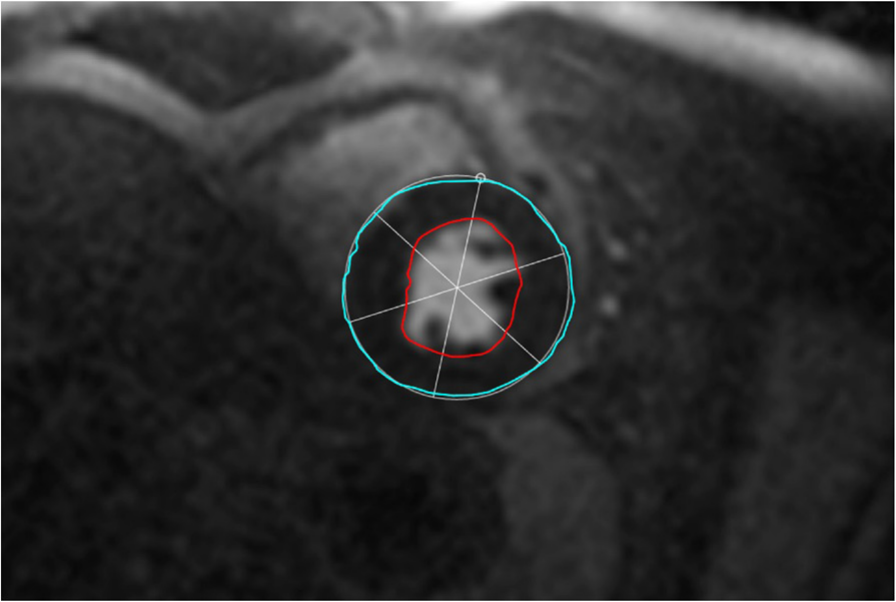
**Segmented slice of a perfusion series.** The epi- and endocardium are here colored in cyan and red respectively. The circumcircle is estimated based on three points on the epicardium. Here, the first point, indicated by a little circle, is co-located with the anterior RV insertion point forming the basis for consistently dividing the myocardium into sections of equal sizes.

The segmentations are given as XML files that can be read and written by the segmentation software [22] as well as the related software in the toolkit for gray scale medical image analysis (MIA) [23] used here to run the experiments described below. The validating XML-scheme is provided with the data, for an example XML file see Listing Listing 1; an example of a segmented slice is given in Figure 2.

#### 

##### Listing 1

Elements of a segmentation set. The work set consists of a description where RV and LV peak enhancement frame can be stored, and a number of images (frames). For each frame, the *star* defined by its center and rays is defined with the first ray passing through the RV insertion point. Then the segmentation sections are given as closed lines of the endo- and epicardium. 

Specifically, the images of each study are separated into three segmentation sets (0 – apical, 1 – middle, and 2 – basal slice). For each frame, that is for each slice and time step of a study set, the following features were segmented: The epi-and endocardium are outlined, and with three points the circumcircle of the LV including the myocardium is identified (Figure 2). The first of these three points is co-located with on of the two RV insertion points (anterior or posterior; consistently selected over the whole image series), thereby making it possible to consistently divide the myocardium into sections for further analysis.In some frames, especially in the pre-contrast phase tissue boundaries can hardly be identified because of missing intensity gradients, for an example see Figure 1a. Here, for consistency of the data (i.e. two contours per slice) a segmentation is guessed.

This has two implications: Firstly, validation based on overlap and boundary distance measures can not be applied. Secondly, consider the automatic evaluation of a time-intensity curve for a myocardical section: Here, a mask taken from one manually selected frame is applied to all images to evaluate the corresponding average intensities. This mask must stem from a properly segmented frame, since the mask should only cover the myocardium in *all* images of a series. On the other hands, for the evaluation of the Ground Truth time-intensity curve, each mask is only used for its corresponding frame. Since the intensities are evaluated as averages over the enclosed regions, an error in the outlining of such a region of homogeneous intensities is of no consequence to the value of this intensity average. Hence, the correct Ground Truth time-intensity curve can be obtained despite the segmentation in some frames not being anatomically correct. Considering that the perfusion analysis measure focuses on local intensity changes in the myocardium, basing the validation of motion compensation methods on only these time-intensity curves is a viable approach.

## Analyses

Two distinct experiments were executed: Firstly, motion compensation was applied to the data sets 1–5 and 7–10 acquired under rest and stress by using the algorithms QUASI-P [13], and ICA-SP [20]; the latter with the enhancements as described in the Methods section. Secondly, both algorithms where applied to the motion-free data set *6-rest* in order to analyze how the algorithms preserve this initially motion-free data. To run the experiments we used the implementation provided with MIA [23].

The parameters for running both methods were set similar to [20], that is, with QUASI-P a gradient decent method was used for optimization (start step size 0.01, stopping condition epsilon 0.01).

For ICA-SP the optimization of the objective function was achieved using the *rank-1 method of the shifted limited-memory variable metric algorithm*[24] (breaking conditions: maximum of 300 iterations, or 0.001 absolute x-tolerance, or 0.001 relative objective function value).

The independent component analysis in ICA-SP uses FastICA [25], first run in deflation mode. If this did not result in a usable signal separation, the symmetric mode was run with a maximum of 400 iterations and the result was used regardless of the convergence of the algorithm. The discrete wavelet transform used the centered Daubechies wavelet family of maximum phase with five vanishing moments [26]. In addition to the sanity checks applied to the LV segmentation, we added a criterion based on the distance between gravity centers of the obtained RV and LV regions. In consideration that the heart of an adult human is approximately 90 mm across in the short axis view, a segmentation was rejected if this distance of the gravity centers was below 30 mm. To account for the higher heart rate in stress studies we added a fall-back check to estimate the independent component (IC) corresponding to motion: If the detection of a motion component from the IC mixing curves based on the wavelet analysis failed we tested whether the mean frequency of one of the curves was above 14 breaths per minute.

The remaining registration parameters are given in Table 2.

**Table 2:** Registration parameters

**Method**	**QUASI-P**	**ICA-SP**
Regularization weight *κ*/scale	0.1 / -	10 / 0.5
Knot spacing/scale	5 / -	16 / 0.5
Multi-resolution-levels	3	3
Passes	1	≤5

“Scale” refers to the value used to scale the according parameter with each new registration pass.

The results reported here were all obtained running the motion compensation in a virtualized environment running an Ubuntu Linux 14.04 (i386) installation, and the version 2.2.0 of MIA (for details see the Additional file 1: Supplementary material).

As validation measures we compared automatically obtained time-intensity curves to manually acquired ones before and after registration. In all cases, the LV myocardium was separated into 12 sections and the key frame mask for the automatic time-intensity curve was obtained from the image marked as constituting peak LV enhancement, because in this image the myocardium can be segmented quite accurately. In the statistical measures one data point is defined by the patient, slice location, study, and its myocardial section observed.

## Experiment 1 - compensation of free breathing motion

In the first experiment, motion compensation for the rest and stress studies of nine patients was run. For 16 of the 54 considered slice series the segmentation of the region of interest around the left ventricle was rejected when running ICA-SP. For these slices the motion compensation had to be run on the full image resolution. For two slices (patient 7, middle, basal) the ICs corresponding to motion could only be identified based on the mean frequency of their mixing curves. However, in these cases the mean frequency for RV enhancement mixing curve was also very close to the chosen threshold, indicating that this approach – here only used as fallback – may not be reliable. Still, motion compensation by applying ICA-SP could be achieved for all series. QUASI-P, on the other hand, failed for one series (patient 7, stress, basal), but was applied successfully for the remaining data.

The obtained validation measures are summarized in Tables 3 and 4. For completeness we not only report the summary results for QUASI-P including all series, but also the results obtained when ignoring the failed series (dubbed QUASI-P ^∗^ in the tables).

**Table 3:** NMSE before and after registration (smaller is better)

**All studies**					
	**Mean**	**Variation**	**Median**	**Min**	**Max**
Unregistered	0.66	0.56	0.51	0.04	4.21
QUASI-P	0.81	1.82	0.41	0.04	16.76
QUASI-P ^∗^	0.61	0.68	0.40	0.04	6.81
ICA-SP	0.52	0.53	0.35	0.04	4.40
**Rest studies**					
Unregistered	0.67	0.59	0.52	0.04	4.21
QUASI-P	0.60	0.63	0.40	0.04	4.57
ICA-SP	0.48	0.51	0.33	0.04	4.35
**Stress studies**					
Unregistered	0.66	0.54	0.49	0.06	3.86
QUASI-P	1.02	2.47	0.42	0.05	16.76
QUASI-P ^∗^	0.62	0.71	0.40	0.05	6.81
ICA-SP	0.56	0.55	0.38	0.05	4.40

Both algorithms result in a significant improvement of the measures, and as with *R*^2^ ICA-SP providing the better motion compensation according to the obtained measurements.

**Table 4:** Pearsons correlation coefficients before and after registration (larger is better)

**All studies**					
	**Mean**	**Variation**	**Median**	**Min**	**Max**
Unregistered	0.82	0.20	0.89	-0.09	1.00
QUASI-P	0.90	0.16	0.95	0.00	1.00
QUASI-P ^∗^	0.91	0.12	0.95	0.13	1.00
ICA-SP	0.92	0.13	0.97	0.17	1.00
**Rest studies**					
Unregistered	0.81	0.18	0.88	0.15	0.99
QUASI-P	0.90	0.13	0.95	0.13	1.00
ICA-SP	0.93	0.11	0.97	0.33	1.00
**Stress studies**					
Unregistered	0.82	0.22	0.91	-0.09	1.00
QUASI-P	0.90	0.19	0.96	0.00	1.00
QUASI-P ^∗^	0.92	0.11	0.96	0.15	1.00
ICA-SP	0.92	0.15	0.97	0.17	1.00

Both algorithms result in a significant improvement of the measures with ICA-SP providing the better motion compensation than QUASI-P according to the obtained average value of *R*^2^.

In all cases (rest, stress, summary) ICA-SP provided better results with respect to the normalized mean square error (NMSE) (Table 3). When considering only the rest studies and the summary statistics this also holds for Pearsons correlation coefficient *R*^2^ (Table 4). For the stress studies, QUASI-P and ICA-SP provide equal mean values when only the slices are taken into account for which motion compensation was successfully executed. These findings confirm that on average ICA-SP performs better than QUASI-P as reported in [20].

## Experiment 2 - no motion

When running the two motion compensation algorithms for a data set that does not exhibit motion to begin with, then ICA-SP is a clear winner over QUASI-P as can be seen from the resulting validation measures given in Table 5.

**Table 5:** Validation measures before and after registration of the motion free series

***R***^**2**^					
	**Mean**	**Variation**	**Median**	**Min**	**Max**
Unregistered	1.00	0.00	1.00	1.00	1.00
QUASI-P	0.50	0.32	0.63	0.00	0.87
ICA-SP	0.94	0.09	0.99	0.66	1.00
**NMSE**					
Unregistered	0.00	0.00	0.00	0.00	0.00
QUASI-P	2.21	1.94	1.37	0.20	6.85
ICA-SP	0.13	0.11	0.11	0.01	0.46

While ICA-SP mostly preserves the data, the application of QUASI-P brings the original alignment to naught.

ICA-SP introduces some small errors, because the algorithm is designed to run at least one registration pass, even if no motion is detected.

QUASI-P on the other hand relies on detecting a sub-set of images that is already well aligned, but doesn’t set a maximum temporal distance between images that are added to the sub-set. Consequently, only very few images are added to this subset, and in its extreme, for the apical slice the estimated pre-aligned subset only consists of the first and the last image of the series. As already reported in [13] the linear interpolation used to create synthetic images for the second registration step is hardly able to model the fast intensity change at the beginning of the series. Here this problem is moved to a new level: Since very few images are included in the pre-aligned subset they simply do not form a sufficient base to model the intensity change of the whole series.

## Discussion

We presented free breathing acquired myocardial perfusion data sets from 10 patients evaluated to have a stress perfusion defect based on cardiac MR myocardial perfusion imaging. By applying algorithms published elsewhere we showed how this data can be used to analyze and validate breathing motion compensation methods. Specifically, we were able to confirm earlier findings, that is, ICA-SP performs better then QUASI-P when applied to free breathing acquired perfusion data with respect to the time-intensity curve based validation measures *R*^2^ and normalized mean square error (NMSE). We were also able to show that ICA-SP is stable considering the preservation of data if no motion is present in a series.

By providing not only the data, but also a full installation of the test bed used to run the experiments as a virtual machine we enable third parties to easily replicate the experiments, to extend the method, and also to run the experiments on their own data. By managing the data and the manual segmentations used for the validation in a public version control system (i.e. Git) it is easy to add new data, and to update and refine segmentations with a proper audit route. We encourage researchers to add their own annotations to the data set, either to provide the possibility to add inter-observer comparisons of segmentations to the presented analysis, or to make the use of the data in other applications possible, for instance, for the validation of segmentation algorithms.

## Methods

Two methods [13,20] are applied to the data to demonstrate its usefulness for the rating and validation of motion compensation algorithms. For both methods software implementations have been made publicly available [23]. For completeness, we give a short description of both algorithms and the validation approach used in this paper.

### Motion compensation exploiting quasi-periodicity of free breathing (QUASI-P)

The algorithm presented in [13] consists of three steps: Firstly, by using normalized gradients fields (NGF) based image similarity measure [27], a global reference image and a subset of images that belong to the same breathing phase are obtained. These images are then non-linearly registered by optimizing NGF. In the second registration step, synthetic reference images are created from the now registered subset that exhibit similar intensity distributions like the original, still unregistered images, but are (ideally) free of motion. Finally, the unregistered images are non-linearly registered to their synthetic reference counterparts by optimizing the sum of squared differences (SSD).

### Motion compensation with an ICA-Wavelet based classification (ICA-SP)

The method presented in [20] is based on an independent component analysis (ICA) of the perfusion series as proposed in [16]. First, an ICA is run on the image series to obtain feature images – the ICs – and a feature mixing matrix. By using a wavelet analysis of this feature mixing matrix, key components of the perfusion series such as motion, and possibly RV and LV enhancement curves are identified. The motion compensation is then achieved by creating synthetic reference images by recombining all ICs but the component identified as motion. This results in a series of reference images that is free of motion but exhibits an intensity profile similar to the original series which makes the application of SSD possible for the following non-linear registration step, where the original images are registered to their synthetic reference counterparts. Since the reference images are quite blurry initially, a multi-pass scheme is applied to achieve full motion compensation. Note, that with this scheme, the rest position is the mean of the breathing movement range, and hence, usually all images of the series are altered by the registration.

If properly identified, the RV and LV IC images can be used to segment the RV and LV cavities, and consequently a region of interest around the LV myocardium can be identified and can then be used to limit motion compensation to that region, thereby also speeding up the calculations. If only non-linear registration is to be applied, this segmentation step is not required.

In order to make result of the LV segmentation more reliable as compared to [20], the segmentation is rejected if the distance between the geometric centers of the estimated RV and LV masks is smaller than a preset value. If the segmentation is rejected the non-linear registration based motion compensation is applied to the full image domain.

Another enhancement to the work presented in [20] is attributed to the identification of ICs presenting motion. In [20] we rely on the observation that the resting heart rate is approximately 75 beats per minute, and the rest breathing rate is about 12 breaths per minute [28]. Since the perfusion imaging is triggered at heart beats, this results in one breathing cycle covering approximately six frames in the time series. However, in stress studies the heart rate is considerably higher and the breathing rate doesn’t necessarily increases proportionally. Hence, in stress studies one breathing cycle may stretch over more heart beats and consequently cover more frames in the image series which results in a failure of the wavelet based labeling method described in [20] to identify ICs that present motion. Since the original image data provides the acquisition times for each frame, ICs corresponding to motion can be identified by thresholding based on the mean frequency of the IC mixing curves representing the breathing rate relative to the heart beat rate. This method of motion identification will only be used as a fallback, when the identification by using the wavelet based method fails.

### Validation

We base the validation solely on the time-intensity curves of the sections of the myocardium that are extracted from the segmentations provided with the given data sets. Given the segmentation of the myocardium obtained from the outlined endo- and epicardium, the LV center point *LV*_c_, and the ray passing from this center point to the marked RV insertion point *RV*_ip_, the myocardium is separated into 12 sections by clock-wise rotating this ray $\vec{{\text{LV}}_{\text{c}}{\text{RV}}_{\text{ip}}}$ with equal angular increments of 30 degree. The result of this separation is equivalent to the separation of six sections shown in Figure 2.

The time-intensity curves are (1) evaluated directly from the segmented data *K*_gt_ (Ground Truth), and by propagating the myocardial section masks obtained from the chosen key frame, (2) over the original image series *K*_org_, and (3) over the image series that was corrected for motion *K*_reg_. In the second case, the section mask of the key frame is used unaltered. In the third case, the section mask is adjusted to the registered key frame according to the transformation that was obtained for motion compensation. Note, that in this case a failed motion compensation may result in a transformed mask that doesn’t contain any pixel for one or more sections. To quantify the registration quality, we evaluate Pearsons correlation coefficient *R*^2^ and the NMSE between the manually obtained time intensity curves *K*_gt_ and the pre-and post registration curves *K*_org_, and *K*_reg_ respectively. Better registration is indicated by a higher correlation *R*^2^ and a lower NMSE. If one of the transformed section masks did not contain any pixel, the correlation *R*^2^ was set to zero to indicate the failure in motion compensation.

Note, that the clinical relevant quantification of the myocardial blood flow (MBF) could also be used as an additional validation measure. However, on one hand, the result of such a quantification is dependent on the used quantification model (e.g. [29,30]), and on the other hand the presented data is not corrected for intensity inhomogeneities, and the motion compensation methods used here do not include such correction. Consequently, using MBF would add an additional layer of evaluations that in itself need validation. Therefore, and since this paper focuses on the presentation of the data and the motion compensation methods are included mostly to illustrate the utility of the data, we refrained from adding MBF as validation measure.

## Availability of supporting data

A static snapshot of the data is provided in the GigaScience data base [31]. The data distribution also includes all the scripts required to run these experiments. In addition, a full installation of the test bed is provided as a virtual hard disk based on a minimal installation of Ubuntu Linux 14.04 (i386). For a detailed description of the data layout and to clarify the licenses of different software in the virtual machine the reader is referred to the Additional file 1: Supplementary material.

In addition, to allow updates and additions to the annotations, the data and scripts are also made available in a public Git repository at https://sourceforge.net/p/tabseg/myoperfdata/ci/master/tree/, in conjunction with a Android based segmentation software [22].

## Abbreviations

IC: Independent component; ICA: Independent component analysis; ICA-SP: Motion compensation by using independent component analysis and spline based non-linear registration; LV: Left ventricle; MIA: Toolkit for gray scale medical image analysis; MRI: Magnetic resonance imaging; NMSE: Normalized mean square error; QUASI-P: Motion compensation exploiting quasiperiodic breathing motion; *R*^2^: Pearsons correlation coefficient; RV: Right ventricle; SR-FLASH: Slew rate – fast low angle shot.

## Competing interests

The authors declare that they have no competing interests.

## Authors’ contributions

GW is responsible for the implementation of the methods, the manual segmentations, and running the experiments, PK provided the data and the medical expertise for the analysis provided here. Both authors read and approved the manuscript.

## Supplementary Material

Additional file 1 Supplementary material. Click here for file
