# Supplementary material for: Free breathing myocardial perfusion data sets for performance analysis of motion compensation algorithms
Source: Gigascience. 2014 Nov 11;3:23. doi: 10.1186/2047-217X-3-23 (PMC4226922; doi:10.1186/2047-217X-3-23)
Supplement: Additional file 1 — Supplementary material. [file 2047-217X-3-23-S1.pdf]

Free Breathing Myocardial Perfusion Datasets for Performance Analysis of Motion Compensation Algorithms
Supplement

Gert Wollny[1,2] and Peter Kellman[3]
[1] Biomedical Imaging Technologies, ETSI Telecomunicación,
Universidad Politécnica de Madrid, Avenide Complutence 30, 28040 Madrid, Spain,
[2] Ciber BBN, Zaragoza, Spain,
[3]Laboratory of Cardiac Energetics, National Heart,
Lung and Blood Institute, National Institutes of Health, DHHS, Bethesda, MD, USA,

1 Supplemental data files

To complement the article, three data files are provided as accompanying material:

- *GigasciencePerfusionTestbedVM.zip* - the test bed including all the data and software needed to run the experiments presented. A description on how to use it is given in section 2.2.
- *mia-2.2.0.tar.xz* - the source code version of the MIA software [1] used to run the experiments.
- *gigascience-myoperfdata.zip* - the data and scripts to run the experiments, for its description see section 2.3.

2 The test bed

2.1 Description

The test bed is provided as a virtual hard disk in VMDK format [2] of 20Gb size (dynamically allocated), comprising a minimalist Ubuntu Linux 14.04 (i386) [3] installation, and the software, data, and scripts required to run the experiments presented in the main article. The installation has been tested with VirtualBox 4.2.22 [4] (Gentoo Linux, 64 bit, AMD FX(tm)-6300), VirtualBox 4.3.6 (GNU Debian/Jessie 64 bit, Intel Core 2 Duo), and VirtualBox 4.3.14 (MS Windows 7, 64 bit, AMD FX(tm)-6300). While the following description on how to run the virtual test bed refers to VirtualBox, other platform virtualization software packages may also be used to run the test bed. Obviously, these alternatives must support the VMDK virtual hard disk format and support Linux i386 as guest operating system.

2.2 Creating and running the virtual machine using VirtualBox

In all cases, the file providing the test bed must be unpacked:

```
unzip GigasciencePerfusionTestbedVM.zip
cd GigasciencePerfusionTestbedVM
```

2.2.1 Setup using the VirtualBox GUI

In order to set up the virtual machine manually by using the VirtualBox GUI the following steps have to be taken: First create a new virtual machine from the menu Machine|New with the following parameters:

- Name and operating system
  - Name: <at your liking>
    e.g. "GigaSciencePerfusionTestbed"
  - Type: Linux
  - Version: Ubuntu (32 bit)
- Memory size: $\geq$ 1024 MB
- Hard drive: (·) *Use an existing hard drive*, select the file *perfusion-with-results-ubuntu-1404-32.vmdk*,

and set the following parameters in the settings panel of the newly created machine:

- System|Motherboard: Enable IO APIC
- System|Processor: Enable PAE/NX
- Network|Adapter 1: NAT

If your host system comprises a multi-core CPU, one might consider to set the number of processors to a value larger then one on the System|Processor panel. With this configuration, the virtual machine can be executed from the VirtualBox GUI and will provide a console. One can log into this console as user *motion* with password *motion*.

To run the machine headless, Host-only networking must be enabled in VirtualBox (File|Preferences|Network) with the DHCP server enabled, and the second network adapter must be enabled as Host-only adapter in the machine specific

network settings panel. When the machine is start up and assuming the VM is named GigaSciencePerfusion-Testbed the IP address can be acquired by running the command

```
vm=GigaSciencePerfusionTestbed
VMIP= $(VBoxManage guestproperty get \
   "$vm" "/VirtualBox/GuestInfo/Net/1/V4/IP")
```

and one can connect then to the machine by using any secure shell client software, e.g. ssh:

```
ssh -l motion $VMIP
```

2.2.2 GNU/Linux as host system

On GNU/Linux, the scripts provided alongside the virtual hard disk can be used to create and run the machine. Specifically, in order to create the virtual machine named *GigaSciencePerfusionTestbed*, one can run the script `./create-vm.sh` from within the unpacked folder. After this script has been run successfully, the virtual machine can be started by running

```
./start-and-connect.sh
```

If successful, the virtual machine will boot up and then prompt for a login as user *motion*; the password is *motion*.

2.2.3 Mac OSX as host system

Given that Mac OSX comprises a fully certified UNIX system, it should be possible to create and run the virtual machine just like with GNU/Linux as a host system. Note, however, that the authors did not have access to a Mac OSX system and that tests run by the reviewers indicate that the machine might be created but the host-only based networking is not properly set up.

2.2.4 Providing a GUI environment within the virtual machine

In order to provide a more familiar graphical environment when running the virtual machine, like, e.g. xfce [5], one can run the following commands within the console of the running virtual machine (cf. [6]):

```
sudo apt-get update
sudo apt-get install xfce4 \\
        virtualbox-guest-x11
sudo reboot
```

Running these commands will require internet access. In this case one might want to review the display settings for the virtual machine, specifically the amount of video memory should be increased, and a *Remote Display* could be enabled. Note, however, that the experiments have to be run from a command line shell.

2.3 Data layout

The data is provided in the home directory of the user *motion* in the folder *myoperfdata*. In the root of this folder various scripts can be found to run motion compensation experiments. Then, for each patient, two folders *StudyN_TYPE* are available, N referring to the number of the patient, and *TYPE* indicating whether this is a *rest* or a *stress* study. Within the study folder the images are given as consecutively numbered DICOM and PNG files. The segmentations are provided as XML files, namely

- segment$N$.set – empty segmentation that only lists the files corresponding to the anatomical location $N \in \{0, 1, 2\}$, (0 – apical, 1 – middle, and 2 – basal slice),
- segment$N$-dcm.set – segmentation set referencing the DICOM images, and
- segment$N$-png.set – segmentation set referencing the PNG images.

New segmentations are best started by using the empty sets segment$N$.set. In addition, to the studies in the folder *scripts* various shell and R scripts are provided needed to run the experiments and evaluate the validation measures.

The git repository and the separate archive follow the same layout.

2.4 Running the motion compensation experiments

Within the virtual machine, in the home directory of the user *motion* the folder *myoperfdata* contains the data and the scripts to run the motion compensation experiments. The output of experiments 1 as published is saved in *output-compensation.published* and the corresponding results of experiment 2 in *output-no-movement.published*.

Here, for each input data set *set* run with the algorithmm *algo* and a parameter set $p_1, p{-}_2, ..., p_n$, the motion compensation result is written to the folder

```
set/mia-algo-p_1-p_2-...p_n.
```

Within these folders a file files *curvesX.txt* can be found that contain the obtained time-intensity curves for slice X. For section i column $3i$ contains the time-intensity values before registration, column $3i + 1$ the ground truth values, and column $3i + 2$ the values after motion compensation.

Experiment 1 is executed by running the script *run-ica-quasi-combined.sh*, the results will be saved in the folders *output-compensation*. The statistical analysis will be stored in the the files *awked-** in a LaTeX friendly table format. Note, that for the stress and summary statistics, two result rows will be presented for the unregistered data, one that includes the series for which registration failed, and one that includes that series. In the paper the latter values that include all series were used to indicated R^2 and NMSE for the unregistered series.

Experiment 2 is executed by running the script *run-ica-quasi-no-movement.sh* and the results will be

saved in the folders *output-no-movement*. The statistical analysis representing Pearsons correlation coefficient will be stored in the file *cc-Rest-all-stat-awked.txt*, and MNSE in *mnse-Rest-all-stat-awked.txt*.

To execute both experiments the script *run-experiments.sh* can be run.

3 Considerations when setting up the test bed from scratch

3.1 Preparing the test bed

The simplest option to install a test bed from scratch is to base the installation on Ubuntu Linux 14.04 like it is done with the virtual test bed. In addition to a basic Ubuntu Linux installation, *tcsh*, the statistical package *R*, and the package *mia-tools* are required to run the presented experiments, and the packages *gnuplot* and *transfig* are required in order to run the *extra-** scripts. Note however, that the discussed enhancements to ICA-SP, namely testing for the RV-LV center distance to test the segmentation and the additional frequency test are not yet provided by the version of MIA that is curently availabe from the official Ubuntu repositories. Instead the latest stable MIA version should be obtained from the authors personal packaging archive (PPA) [7]. The software package versions relevant for the calculations used in the test bed are given in Table 1, all packages were build according to the Ubuntu packaging scripts.

Table 1: Relevant software versions

Software	Ubuntu Package	Version
BLAS	libblas3	1.2.20110419-7
FFTW	libfftw3-3	3.3.3-7ubuntu3
GSL	libgsl0ldbl	1.16+dfsg-1ubuntu1
IT++	libitpp8	4.3.1-2
MIA	mia-tools	2.2.0-1trustymia4
NLOpt	nlopt0	2.4.1+dfsg-1ubuntu1
R	r-base	3.0.2-1ubuntu1

Alternatively, the current development version of MIA can be used, instructions how to compile and install the software are given on-line [8]. In order to run the experiments the optional software packages *NLopt*, *DCMTK*, *IT++*, and *PNG* are required to compile MIA and run the experiments. Note that only POSIX compatible operating systems are supported, and only GNU/Linux running on Intel compatible processors and PowerPC has been tested.

With the software for the testbed in place, the data and scripts can either be acquired from the GigaScience repository, or from the public git repository [9].

4 A note on reproducibility

Although the test bed provides a well defined environment to run the experiments, various runs may result in silghtly different results, because FastICA uses random numbers for initialization. When running the software on different machines, other factors have to be taken into account: For example, older versions of IT++ have a bug and MIA will not utilized the deflation approach of FastICA if at compile time such an older version of this library is detected. Second, MIA makes use of a lot of third party libraries that may also come in different versions and may have been compiled with different compiler flags.

Also note, that depending on the processor architecture and the optimization level results of floting point operations may vary slightly. For instance, when running software compiled for i386, floating point operations rely on the old i87 instructions set. Here floating point numbers are represented with 80 bit in the processor registeres, and only when the value is written to the working memory, the accuracy is reduced to 32 bit for single floating point values or 64 for double floating point values. Hence, since with a higher optimization level usually more operations are executed without storing intermediate values in working memory, rounding errors will differ from results that are obtained by running the same code compiled with a lower optimization level that issues code that stores more intermediate results, and hence maintains a lower accuracy on these intermediate results. Likewise, when switching from i386 to the amd64 architecture, the compiler will issue SSE instructions instead of i87 instructions, and more register are available to store intermediate results, hence, results might also differ slightly when running the code on these two different architectures (cf. [10]).

To illustrate these issues, in the appendix of this document we report additional results for experiment 1 that were obtained on different versions of GNU Linux and with a different hardware than used in the main document. In these two cases a pre-release version of MIA was used (branch: master, tag: gigascience-submission). Nevertheless, although the numbers vary slightly, the actual results – that ICA-SP performs better than QUASI-P – holds.

4.1 Closing note

Public forums for discussing the software and data are available at sourceforge.net [11, 12].

Appendix

Results obtained on Gentoo 64 bit

- Processor: AMD FX(tm)-6300
- Compiler flags: -O2 -g -funroll-loops -ftree-vectorize -pthread -march=native -mtune=native
- IT++: Ebuild version 4.2-r1
- NLopt: 2.2.4
- BLAS implementation: ATLAS

Table 2: Pearsons Correlation coefficients before and after registration (larger is better).

All studies

	mean	variation	median	min	max
unregistered	0.82	0.20	0.89	-0.09	1.00
QUASI-P	0.90	0.16	0.95	0.00	1.00
QUASI-P*	0.91	0.12	0.95	0.16	1.00
ICA-SP	0.92	0.13	0.97	0.15	1.00

Rest studies

unregistered	0.81	0.18	0.88	0.15	0.99
QUASI-P	0.90	0.13	0.95	0.16	1.00
ICA-SP	0.93	0.11	0.97	0.34	1.00

Stress studies

unregistered	0.82	0.22	0.91	-0.09	1.00
QUASI-P	0.90	0.19	0.96	0.00	1.00
QUASI-P*	0.92	0.11	0.96	0.16	1.00
ICA-SP	0.91	0.16	0.97	0.15	1.00

Table 3: NMSE before and after registration (smaller is better).

All studies

	mean	variation	median	min	max
unregistered	0.66	0.56	0.51	0.04	4.21
QUASI-P	0.81	1.82	0.41	0.04	16.76
QUASI-P*	0.61	0.68	0.40	0.04	6.92
ICA-SP	0.53	0.56	0.35	0.03	5.02

Rest studies

unregistered	0.67	0.59	0.52	0.04	4.21
QUASI-P	0.60	0.63	0.40	0.04	4.69
ICA-SP	0.48	0.49	0.32	0.03	3.86

Stress studies

unregistered	0.66	0.54	0.49	0.06	3.86
QUASI-P	1.02	2.48	0.42	0.05	16.76
QUASI-P*	0.62	0.73	0.40	0.05	6.92
ICA-SP	0.58	0.61	0.39	0.04	5.02

Results obtained on virtualized GNU Debian/Linux 64, Jessie

- Processor: AMD FX(tm)-6300
- Compiler flags: -O3 -DNDEBUG
- IT++: Package version 4.3.1-2
- NLopt: 2.4.1+dfsg-1
- BLAS implementation: blas (netlib)

Table 4: Pearsons Correlation coefficients before and after registration (larger is better).

All studies

	mean	variation	median	min	max
unregistered	0.82	0.20	0.89	-0.09	1.00
QUASI-P	0.90	0.16	0.95	0.00	1.00
QUASI-P*	0.91	0.12	0.96	0.15	1.00
ICA-SP	0.93	0.12	0.97	0.17	1.00

Rest studies

unregistered	0.81	0.18	0.88	0.15	0.99
QUASI-P	0.90	0.13	0.95	0.16	1.00
ICA-SP	0.93	0.11	0.97	0.33	1.00

Stress studies

unregistered	0.82	0.22	0.91	-0.09	1.00
QUASI-P	0.90	0.19	0.96	0.00	1.00
QUASI-P*	0.92	0.11	0.96	0.16	1.00
ICA-SP	0.93	0.13	0.97	0.17	1.00

Table 5: NMSE before and after registration (smaller is better).

All studies

	mean	variation	median	min	max
unregistered	0.66	0.56	0.51	0.04	4.21
QUASI-P	0.81	1.82	0.41	0.04	16.76
QUASI-P*	0.61	0.68	0.40	0.04	6.92
ICA-SP	0.50	0.47	0.35	0.03	4.33

Rest studies

unregistered	0.67	0.59	0.52	0.04	4.21
QUASI-P	0.60	0.63	0.40	0.04	4.71
ICA-SP	0.48	0.51	0.33	0.03	4.33

Stress studies

unregistered	0.66	0.54	0.49	0.06	3.86
QUASI-P	1.02	2.47	0.42	0.05	16.76
QUASI-P*	0.62	0.71	0.41	0.05	6.89
ICA-SP	0.51	0.43	0.37	0.04	2.78

References

[1] https://mia.sourceforge.net (2014-04-06)

[2] https://www.vmware.com/support/developer/vddk/vmdk_50_technote.pdf?src=vmdk (2014-04-06)

[3] http://www.ubuntu.com (2014-09-05)

[4] https://www.virtualbox.org/ (2014-04-06)

[5] http://www.xfce.org/ (2014-08-27)

[6] https://www.debian.org/doc/user-manuals (2014-08-27)

[7] https://launchpad.net/~gert-die/+archive/ubuntu/trusty-mia(2014-09-05)

[8] https://sourceforge.net/p/mia/wiki/Installation%20on%20Unix-like%20operating%20systems/ (2014-08-27)

[9] https://sourceforge.net/p/tabseg/myoperfdata/ci/master/tree/ (2014-04-07)

[10] http://software.intel.com/en-us/forums/topic/309695(2014-04-06)

[11] https://sourceforge.net/p/mia/discussion/(2014-04-07)

[12] https://sourceforge.net/p/tabseg/discussion(2014-04-07)
